# Understanding the barriers and facilitators of COVID-19 vaccine hesitancy amongst healthcare workers and healthcare students worldwide: An umbrella review protocol

**DOI:** 10.1371/journal.pone.0283897

**Published:** 2023-03-31

**Authors:** Jemma McCready, Dania Comparcini, Bethany Nichol, Mary Steen, John Unsworth, Marco Tomietto

**Affiliations:** 1 Faculty of Health and Life Sciences, Department of Social Work, Education and Community Wellbeing, Northumbria University, Newcastle upon Tyne, United Kingdom; 2 Politecnica Delle Marche University of Ancona, Ancona, Italy; 3 Azienda Ospedaliera Universitaria “Ospedali Riuniti” di Ancona, Ancona, Italy; 4 Department of Social Work, Education, and Community Wellbeing, Northumbria University, Newcastle upon Tyne, United Kingdom; 5 Faculty of Health and Life Sciences, Department of Nursing, Midwifery and Health, Northumbria University, Newcastle upon Tyne, United Kingdom; 6 Research Unit of Nursing Science and Health Management, University of Oulu, Oulu, Finland; 7 Universita’ degli Studi di Bari “Aldo Moro”, Bari, Italy; Xiamen University - Malaysia Campus: Xiamen University - Malaysia, MALAYSIA

## Abstract

Healthcare workers (HCWs) and healthcare students are at increased risk of becoming infected with and being a vector of transmission of COVID-19. Vaccination efforts amongst this group of persons have been hampered in some countries by hesitancy to uptake the COVID-19 vaccine. The factors related to vaccine hesitancy have been reported in several systematic reviews. However, a comprehensive overview of barriers and facilitators of COVID-19 vaccine hesitancy is greatly needed to address effective interventions in this population. Understanding and designing effective strategies to promote vaccination among HCWs is pivotal to secure an appropriate and safe healthcare provision. The current protocol describes the methodology for an Umbrella Review that explores the barriers and facilitators of COVID-19 vaccine hesitancy for HCWs and healthcare students. The databases that will be searched are CINAHL, MedLine, Cochrane Library, PubMed, ProQuest, Web of Science, Science Direct, IBSS, Google Scholar, and Epistemonikos. Studies will be eligible for inclusion if they: (i) conducted a systematic review (with or without meta-analysis); (ii) included primary sources utilizing a quantitative methodology; (iii) investigated factors related to COVID-19 vaccine hesitancy; (iv) and included a sub/population of HCWs or healthcare students aged 18–65. The screening processes and data extraction will be conducted independently by two reviewers. The Joanna Briggs Institute (JBI) Critical Appraisal Checklist for Systematic Reviews and Research Syntheses will be used to assess the methodological quality of the included reviews. The degree to which the included reviews contain the same primary studies will also be assessed and reported. The outcomes of this review will have wide-reaching implications for the research area, healthcare systems and institutions, and governments worldwide.

## Introduction

The coronavirus disease 2019 (COVID-19) pandemic has been considered the most pressing global issue impacting every aspect of people’s lives [1]. Globally, as of 17 June 2022, there have been over 535 million confirmed cases of COVID-19 and 6,314,972 deaths [2]. Public interest in vaccines has increased dramatically and at an unprecedented level following the COVID-19 pandemic [3]. To date, current vaccines continue to prevent severe disease and death despite several variants of concern (VOC) that appear to spread faster than the initial SARS-CoV-2 strain [4]. However, different variant-updated vaccines are under clinical development to enhance the magnitude, duration, and breadth of immunity afforded by COVID-19 vaccines to achieve public health impact now and in the future [5]. It is important to note that, nowadays, vaccine uptake tends to decrease globally with each additional recommended dose. Nevertheless, updated vaccines could reverse this trend [5]. In this rapidly evolving scenario, an in-depth understanding of vaccine hesitancy with a focus on the development of future, more effective strategies to promote adherence to vaccination remains a priority.

The pandemic also affected healthcare workers (HCWs) worldwide impacting on healthcare provision through staff absence and illness. Vaccination among HCWs and healthcare students with access to clinical settings is pivotal to both providing HCWs and students’ safety and patient safety. Previous research demonstrated higher mortality rates in clinical settings where employees’ vaccine uptake was lower [6]. However, historically, HCWs are reluctant to get vaccinated, when considering the seasonal influenza vaccination campaigns: from 2015 to 2018 the European uptake was below 40% [7,8]. Different countries adopted different policy approaches to HCWs’ vaccination: from a recommendation, to a legal requirement, to suspension from clinical practice [9]. Understanding and designing effective strategies to promote vaccination among HCWs is pivotal to secure an appropriate and safe healthcare provision.

Vaccine hesitancy is a complex phenomenon, defined as “a delay in acceptance or refusal of vaccination despite availability of vaccination services” [10]. The reasons for vaccine hesitancy include environmental factors (public health policies, social factors, and media messages), agent factors (the individual perceived susceptibility to the disease in addition to the perception of the safety and efficacy of the vaccine) and the host factors (individual’s knowledge, previous experience, educational and income levels). In addition to this epidemiological triad of factors, it is important to consider the variability that exists between different countries and regions of the world [11] and population groups (e.g., general adults, pregnant women, university students, healthcare professionals). The analysis of such factors, focusing on subpopulations at increased risk for infection, such as healthcare workers (HCWs) and healthcare students, is needed to address COVID-19 vaccine hesitancy. Due to the scope and magnitude of this pandemic, there is a substantial need to develop and implement intervention strategies that aim to reduce vaccine hesitancy [12,13]. Vaccination is the main strategy to tackle this threat, so maintaining vaccine uptake over time is imperative [14].

In fact, even if the safety and the effectiveness of vaccines against COVID-19 are well established [15], hesitancy and refusal among HCWs [1] and healthcare students [16] to receive a COVID-19 vaccine remain a major concern for several reasons. Firstly, HCWs and healthcare students are at an increased risk of becoming infected with and being a vector of coronavirus transmission due to their direct contact with clinically vulnerable patients in the workplace [17]. Secondly, vaccination recommendations from healthcare professionals strongly determine vaccine willingness in the general population [18]. As trusted sources of information on health problems and prevention, HCWs are ideally placed to recommend the vaccination to others. Therefore, greater vaccine confidence and acceptance in HCWs may lead to increased vaccine acceptance in the general population [19].

Over the last 18 months, there has been a rapid growth of studies exploring factors that contribute to uptake or hesitancy towards the COVID-19 vaccine for HCWs and healthcare students. As a result, numerous systematic reviews and meta-analyses have been conducted to identify the most significant factors associated with vaccine hesitancy in this population. However, there is wide variation in the factors that have been reported as the main determinants of vaccine hesitancy between the systematic reviews, such as demographics (age, gender, professional role) [20–22], previous vaccination history [23], perceptions of vaccine safety and effectiveness [24,25], and trust in government and medical establishments [23,26]. Currently, results from meta-analyses provide contradictory evidence. For example, age and gender were significant determinants in one review [27] but not in a review by Geng and colleagues [28]. As intervention strategies are dependent upon this evidence, there is a need for a high-level overview of the topic. An Umbrella Review is a type of evidence synthesis used to summarize the evidence from multiple research syntheses [29], its comparing and contrasting nature will help address the heterogeneous nature of the results from systematic reviews [30]. The results from an Umbrella Review can inform guidelines, clinical practices, and recommendations for future research [31]. Therefore, an Umbrella Review will be undertaken to synthesize evidence on determinants (either as a barrier or facilitator) of COVID-19 vaccine hesitancy in HCWs and healthcare students worldwide and provide some recommendations for intervention.

### Aim and research questions

The aim of this Umbrella Review is to provide broad and complete evidence on the barriers and facilitators of COVID-19 vaccine hesitancy in HCWs and healthcare students by synthesizing the information across systematic reviews.

The research questions addressed in this Umbrella Review are:

- What are the barriers and facilitators associated with COVID-19 vaccine hesitancy in HCWs worldwide?
- What are the barriers and facilitators associated with COVID-19 vaccine hesitancy in healthcare students worldwide?

## Materials and methods

### Design

An Umbrella Review of previous systematic reviews and meta-analyses protocol has been deigned in accordance with the Cochrane guidelines for an overview of reviews [29] and the JBI guidelines for Umbrella Reviews [31]. The study protocol was registered on PROSPERO (registration number: CRD42022327354).

### Inclusion and exclusion criteria

The PEO framework will be used to provide explicit criteria on the participants/population (P), exposure (E), outcomes (O) for inclusion.

#### Participants/Population

Studies that include HCWs or healthcare students aged between 18 and 65 will be included. The review will not exclude based on the gender, ethnicity, or race of participants. The review will be limited to human studies; therefore, animal studies will be excluded.

#### Exposure

Studies exploring COVID-19 vaccine hesitancy will be included. Studies investigating vaccine hesitancy towards other vaccines (e.g., influenza) will be excluded.

#### Outcomes

We will consider, as main outcomes, any variables that are potential determinants, either as a barrier or a facilitator, of COVID-19 vaccine hesitancy. Anticipated determinants include beliefs, attitudes and motivation (e.g. perceived personal risk, anti-vaccination beliefs, emotional factors, preventative health beliefs and behaviours), trust in healthcare systems and vaccine providers (e.g. perceived lack of scientific information), socioeconomic and demographic data (e.g. ethnicity, gender, educational and occupational background), risks and benefits, and vaccine-specific issues (e.g. concerns regarding long-term adverse effects, safety concerns, concerns about the rapid development of the vaccine).

#### Types of studies/study design

This review will include systematic reviews and meta-analyses. In detail, systematic reviews will be included if they will meet the methodological requirements for data extraction and quality appraisal, such as the number of reviewers and the inter-rater agreement procedure, and if they will present the Preferred Reporting Items for Systematic Reviews and Meta-analysis (PRISMA) guidelines to report the results. Meta-analyses will be considered if they will report a clear estimate of the heterogeneity and the overall effect size. Studies that have only conducted systematic reviews without meta-analyses are still eligible for inclusion. Due to the ongoing, fast nature of the topic area, we will also include rapid systematic reviews and scoping reviews. Any reviews incorporating theoretical studies or text and opinion as their primary source of evidence will be excluded. This review will focus on quantitative studies only; therefore, reviews primarily focusing on qualitative and mixed-methods studies will be excluded. With regards to reviews that include both qualitative and quantitative research, only the data referring to quantitative studies will be extracted for this Umbrella Review. Peer-reviewed and pre-printed studies will be included due to the fast nature of the research surrounding the COVID-19 pandemic. Other non-peer-reviewed material will be excluded. Only studies published in English will be considered. Studies published before 2019 will be excluded.

### Search strategy

Initial searches have been performed on PROSPERO, JBI Systematic Review Register, and Open Science Framework, and no pre-registered protocols for an Umbrella Review with similar aims were identified. Before starting the review, the search strategy (Table 1) will be peer-reviewed by a librarian using the Peer Review of Electronic Search Strategies (PRESS) checklist. Changes will be modified accordingly [32]. The initial pre-determined search strings will be entered into the following databases: CINAHL, Cochrane Library, PubMed/MEDLINE, ProQuest, Web of Science, Science Direct, the International Bibliography of Social Sciences (IBSS), Google Scholar, and Epistemonikos. The search strategy will be modified accordingly to fit each database’s requirements. The keywords contained in the title, abstract, and index terms of the retrieved papers will be documented and used to inform additional searches, if necessary. Upon completion of full-text screening, the reference lists of the included reviews will be hand-searched to ensure that any relevant articles were not missed by the search strategy. The steps outlined in the search strategy will be repeated until saturation is achieved.

**Table 1:** Preliminary search terms.

COVID-19 OR COVID19 OR COVID 19 OR SARS-CoV-2 OR SARS-CoV2 OR SARSCoV2 OR SARSCoV-2 OR SARS coronavirus 2 OR 2019 nCoV OR 2019nCoV OR 2019-novel CoV OR nCov 2019 OR nCov 19 OR severe acute respiratory syndrome coronavirus 2 OR novel coronavirus disease OR novel corona virus disease OR corona virus disease 2019 OR coronavirus disease 2019 OR novel coronavirus pneumonia OR novel corona virus pneumonia OR severe acute respiratory syndrome coronavirus 2 OR covid-19 innoculat* OR covid-19 immuni?*	AND	vaccine hesitan* OR vaccine accept* OR vaccine refus* OR vaccine reluct*	AND	healthcare worker OR health professional OR health personnel OR medical staff OR medical student* OR healthcare student* doctor* OR nurs* OR student nurs*

### Selection process

All citations retrieved from the databases will be imported into EndNote (via RIS files) for reference management and duplicate removal. The resulting records will be uploaded onto Rayyan [33], an online data screening and extraction tool, which will be used to facilitate the selection and review process. One reviewer will independently screen the titles and abstracts of all retrieved citations for eligibility against the inclusion criteria. Full texts for the eligible citations will then be retrieved and screened for relevance to the review aim. A second reviewer will independently conduct title and abstract screening and full-text screening on a random allocation of 10% and 25% of citations, respectively. Disagreements will be resolved by the decision of a third reviewer. Inter-rater agreement will be assessed with Cohen’s Kappa and interpreted in accordance with Cohen’s Kappa conventions [34] as follows: k < 0.20 ‘*poor*’, 0.21 < k < 0.40 ‘*fair*’, 0.41 < k < 0.60 ‘*moderate*’, 0.61 < k < 0.80 ‘*good*’, 0.81 < k < 1.00 ‘*very good*’.

### Data extraction

The JBI Data Extraction Form for Review of Systematic Reviews and Research Syntheses will be used to capture the following data: study details (author/year, objectives, participants (characteristics/total number), setting/context, description of exposure, search details (sources searched, range (years) of included studies, number of studies included, types of studies included, country of origin of included studies), appraisal (appraisal instruments used, appraisal rating), and analysis (method of analysis, outcome assessed, results/findings, significance/direction, heterogeneity). Additional types of relevant data may be extracted if they are deemed relevant to the review aims/ objectives by the review team. One reviewer will independently extract the required data from the included articles into the data extraction form created in Excel. Another member of the research team will independently extract data from 10% of the included articles. Both data extraction forms will be reviewed, and a third reviewer will resolve any discrepancies. If the inter-rater agreement (Cohen’s Kappa) will be outside the threshold recommended as satisfactory [34], the percentage for independent extraction will be raised until a satisfactory agreement will be achieved.

### Assessment of methodological quality

The JBI Critical Appraisal Checklist for Systematic Reviews and Research Syntheses [31] will be used to appraise the methodological quality of the reviews included in this Umbrella Review. The 11-item checklist assesses the possibility of bias in three areas of the review: 1) design (i.e., the explicitness of review questions, appropriateness of inclusion criteria and search strategy, adequacy of search sources, and appropriateness of critical appraisal tool); 2) conduct (i.e., minimization of bias during critical appraisal processes and data extraction, and assessment of publication bias); and 3) analysis (i.e., appropriateness of synthesis, support for policy recommendations, and appropriateness of research directives) [31]. The questions are answered using a 4-item response scale *(*‘*yes*,’ ‘*no*,’ ‘*unclear*,’ or ‘*not applicable*’). One reviewer will independently complete the critical appraisal. A second reviewer will also independently assess 10% of the sample. Inter-rater agreement will be assessed with Cohen’s Kappa and interpreted in accordance with Cohen’s Kappa conventions [34].

### Data synthesis

The degree to which the included reviews contain the same primary studies will be assessed using the Corrected Covered Area (CCA) calculation recommended by Pieper and colleagues [35]. The CCA score will be interpreted as follows: 0–5 = ‘*Slight overlap*’; 6–10 = ‘*Moderate overlap*’; 11–15 = ‘*High overlap*’; and >15 = ‘*Very high overlap*’ [35]. As per recommendations, no action will be taken in response to a CCA score that indicates a high level of overlap; instead, this will be discussed as a review limitation.

The data contained within the reviews will be collated, and a descriptive numerical summary and a thematic analysis will be conducted. The numerical summary will describe the characteristics of the included studies in table format. A narrative summary of the key determinants will be provided, which will describe in detail the determinants (barriers and facilitators) of COVID-19 vaccine hesitancy for HCWs and healthcare students. We will describe any subgroup in the two target populations according to the participants’ characteristics considered in the review, such as age, gender, ethnicity, socioeconomic status, educational and occupational background or geographical region.

## Discussion and implications

This review will help to provide a comprehensive understanding of the barriers and facilitators of COVID-19 vaccine hesitancy in both HCWs and healthcare students. The results from this Umbrella Review will have wide-reaching implications for the research area, healthcare systems and institutions, governments worldwide, and individuals themselves. To the researchers’ knowledge, no other published Umbrella Reviews exist on this topic for this population group. This research will inform the policymaker and the healthcare institution to design vaccination campaign among HCWs so as to promote the healthcare systems capability, HCWs and students’ safety and patient safety outcomes.

### Study limitations

There are several limitations of this Umbrella Review that are worthy of discussion at the outset. Firstly, only quantitative data will be included, which may introduce potential biases into the study selection stage of the review. Secondly, other types of reviews will be included, such as scoping reviews and rapid systematic reviews. The researchers acknowledge that this may reduce the quality of evidence in our review; however, it is believed that including a range of reviews will help capture a greater scope of literature and strengthen the outcomes of the review. To account for this, the researchers will critically appraise and report the methodological quality of the reviews included. It is envisaged that there may be potential issues with the repeated inclusion of primary studies across the reviews due to the small timeframe of the topic (2019–2022). Therefore, the researchers aim to assess the degree of overlap between the studies using the Corrected Cover Area statistic and provide a discussion of the finding and their potential impacts on our results in the discussion section of our outputs.

Finally, the search strategy will focus on paper published in English and this could limit the coverage of the literature search and may introduce a bias in our findings.
